# White matter hyperintensities and Alzheimer’s disease susceptibility: a genome‐wide interaction study

**DOI:** 10.1002/alz.084982

**Published:** 2025-01-03

**Authors:** Yuen Yan Wong, Che‐Yuan Wu, Daniel K Mori‐Fegan, Shiropa Noor, Lisa Y. Xiong, Saira S. Mirza, Mario Masellis, Ekaterina Rogaeva, Yutaka Amemiya, Arun Seth, Joel Ramirez, Christopher J.M. Scott, Fuqiang Gao, Sean Symons, Chinthaka Chris Heyn, Katherine Zukotynski, Aparna Bhan, Richard H Swartz, Demetrios J. Sahlas, Sandra E Black, Meghan J. Chenoweth, Walter Swardfager

**Affiliations:** ^1^ Sunnybrook Health Sciences Centre, Toronto, ON Canada; ^2^ University of Toronto, Toronto, ON Canada; ^3^ Sunnybrook Research Institute, Toronto, ON Canada; ^4^ Heart and Stroke Foundation Canadian Partnership for Stroke Recovery, Toronto, ON Canada; ^5^ Tanz Centre for Research in Neurodegenerative Diseases, University of Toronto, Toronto, ON Canada; ^6^ McMaster University, Hamilton, ON Canada; ^7^ University of British Columbia, Vancouver, BC Canada; ^8^ ICES, Toronto, ON Canada; ^9^ Toronto Dementia Research Alliance, Toronto, ON Canada; ^10^ Campbell Family Mental Health Research Institute, Centre for Addiction and Mental Health, Toronto, ON Canada; ^11^ KITE Toronto Rehabilitation Institute, Toronto, ON Canada

## Abstract

**Background:**

White matter hyperintensities (WMH) are commonly observed on MRI in Alzheimer’s disease (AD), but the molecular pathways underlying their relationships with the ATN biomarkers remain unclear. The aim of this study was to identify genetic variants that may modify the relationship between WMH and the ATN biomarkers.

**Method:**

This genome‐wide interaction study (GWIS) included individuals with AD, MCI, and normal cognition from ADNI (n = 1012). WMH were measured from FLAIR MRI using an automated atlas‐based segmentation. CSF Aβ42, tau, and p‐tau concentrations were measured using Roche Elecsys immunoassays. Hippocampal volumes were obtained using FreeSurfer. Interaction effects between single nucleotide polymorphisms (SNP) and WMH volumes on ATN biomarkers were assessed using linear regression models, adjusting for age, sex, diagnosis, MMSE, APOE‐ε4 status, head‐size, and 4 genetic principal components in PLINK2. Linkage disequilibrium‐based clumping (LD: 0.5; physical distance: 250kb) was performed in PLINK1.9, visualized with LocusZoom. Significant SNP‐WMH interactions discovered in ADNI were validated in participants from the Sunnybrook Dementia Study (n = 433) and UK Biobank (n = 16720).

**Result:**

A 30‐SNP intergenic locus on chromosome 18 (lowest p‐value‐SNP: rs72899960 T>A, β = 227.0 pg/ml, SE = 10.2, p = 2.30 × 10^‐8^, MAF = 11.1%, Imputation‐R^2^>0.99%, n = 848) achieved genome‐wide significance interacting with WMH to predict Aβ42, with the nearest gene being a novel long noncoding RNA (lncRNA), *ENSG00000286844* (‐360KB). A 9‐SNP intronic locus on chromosome 11 (lowest p‐value‐SNP: rs3912008 C>T, β = 0.25 cm^3^, SE = 0.044, p = 1.47 × 10^‐8^, MAF = 22.6%, Imputation‐R^2^>99%, n = 987) achieved genome‐wide significance interacting with WMH to predict hippocampal volume, and was found within the microRNA gene *MIR4300HG*. Both SNPs showed additive moderation effects such that associations between WMH and ATN markers in major allele homozygotes were diminished in heterozygotes, and diminished further in minor allele homozygotes.

The interaction between *MIR4300HG‐*rs3912008 and WMH in predicting hippocampal volume replicated in dominant models in both the Sunnybrook Dementia Study (F_1,431_ = 7.98, coefficient = 0.14 cm^3^, SE = 0.05, p = 0.005) and in UK Biobank (F_1,16623_ = 5.32, coefficient = 0.025 cm^3^, SE = 0.011, p = 0.021).

**Conclusion:**

Genomic variants moderate relationships between WMH and ATN biomarkers suggesting a novel regulatory role for a long non‐coding RNA, and the involvement of microRNA *MIR4300HG*. This interaction study may help identify the contribution of specific genes and the potential pathways involved in the relationships between WMH and AD biomarkers.

